# Hospital-based screening to detect patients with cadmium nephropathy in cadmium-polluted areas in Japan

**DOI:** 10.1186/s12199-019-0762-3

**Published:** 2019-01-26

**Authors:** Toru Sasaki, Hyogo Horiguchi, Akira Arakawa, Etsuko Oguma, Atsushi Komatsuda, Kenichi Sawada, Katsuyuki Murata, Kazuhito Yokoyama, Takehisa Matsukawa, Momoko Chiba, Yuki Omori, Norihiro Kamikomaki

**Affiliations:** 1 Department of Internal Medicine, Akita Rosai Hospital, Japan Organization of Occupational Health and Safe, Akita, 018-5604 Japan; 2 Fukunaga Clinic, Akita, 018-5334 Japan; 3 Department of Hygiene, Kitasato University School of Medicine, 1-15-1 Kitasato, Minami-ku, Sagamihara, Kanagawa 010-8543 Japan; 4 Department of Environmental Health Sciences, Akita University, Graduate School of Medicine, Akita, 010-8543 Japan; 5 Department of Environmental and Preventive Medicine, School of Medicine, Jichi Medical University, Tochigi, 329-0498 Japan; 6 Kosaka-machi Clinic, Akita, 017-0202 Japan; 7 Department of Hematology, Nephrology, and Rheumatology, Akita University Graduate School of Medicine, Akita, 010-8543 Japan; 8 Department of Epidemiology and Environmental Health, Juntendo University Faculty of Medicine, Tokyo, 113-8421 Japan; 9 Department of Emergency, Saiseikai Utsunomiya Hospital, Utsunomiya, 321-0974 Japan

**Keywords:** Cadmium, Nephropathy, Screening, Hospital, ß_2_-microglobulin

## Abstract

**Background:**

In health examinations for local inhabitants in cadmium-polluted areas, only healthy people are investigated, suggesting that patients with severe cadmium nephropathy or *itai*-*itai* disease may be overlooked. Therefore, we performed hospital-based screening to detect patients with cadmium nephropathy in two core medical institutes in cadmium-polluted areas in Akita prefecture, Japan.

**Methods:**

Subjects for this screening were selected from patients aged 60 years or older with elevated serum creatinine levels and no definite renal diseases. We enrolled 35 subjects from a hospital in Odate city and 22 from a clinic in Kosaka town. Urinary ß_2_-microglobulin and blood and urinary cadmium levels were measured.

**Results:**

The criteria for renal tubular dysfunction and the over-accumulation of cadmium were set as a urinary ß_2_-microglobulin level higher than 10,000 μg/g cr. and a blood cadmium level higher than 6 μg/L or urinary cadmium level higher than 10 μg/g cr., respectively. Subjects who fulfilled both criteria were diagnosed with cadmium nephropathy. Six out of 57 patients (10.5% of all subjects) had cadmium nephropathy.

**Conclusions:**

This hospital-based screening is a very effective strategy for detecting patients with cadmium nephropathy in cadmium-polluted areas, playing a complementary role in health examinations for local inhabitants.

**Registration number:**

No. 6, date of registration: 6 June, 2010 (Akita Rosai Hospital), and No. 1117, date of registration: 26 December, 2013 (Akita University).

## Background

Cadmium is a toxic heavy metal ubiquitously found in the environment. Humans intake cadmium from food that contains a relatively high amount of cadmium, such as rice, vegetables, seaweeds, and mollusks [1]. After its absorption through the intestines, cadmium mainly accumulates in the kidneys in an age-dependent manner because of its very long biological half-life (15–30 years). Therefore, an excessive cadmium intake leads to the renal over-accumulation of cadmium, which results in renal injury in old age. Cadmium-induced renal injury is a multiple proximal renal tubular dysfunction that is characterized by the excessive urinary excretion of water, glucose, amino acids, low-molecular-weight proteins, calcium, and phosphate, and is called “cadmium nephropathy” [2]. Patients with cadmium nephropathy do not exhibit any significant clinical symptoms except for polyuria [3]. Therefore, patients are often not diagnosed under normal clinical settings unless the disease is suspected and an investigation of renal tubular function, such as the measurement of urinary levels of α_1_-microglobulin (α1MG) or ß_2_-microglobulin (ß2MG), is performed. Glomerular function gradually decreases with the progression of renal tubular dysfunction, leading to elevated serum creatinine levels [2].

There are several cadmium-polluted areas in Japan attributed to slags, waste fluids, or emissions from local mines or smelters [4]. The inhabitants of these areas, particularly farmers, are at risk of excessive oral exposure to cadmium through the life-long consumption of locally produced rice, which results in the development of cadmium nephropathy [5]. The Jinzu river basin area in Toyama prefecture is a representative of these areas, in which many female farmers have “*itai*-*itai* disease,” the clinical manifestation of which is osteomalacia following cadmium nephropathy through the continued excessive renal excretion of calcium and phosphate [2, 6, 7]. Until 2017, 200 patients with *itai*-*itai* disease have been officially acknowledged. Although the excessive exposure of local inhabitants to cadmium stopped after the restoration of polluted rice fields was completed in 2011, it is estimated that new patients with *itai*-*itai* disease as well as cadmium nephropathy will continue to occur in the future. Therefore, official health examinations are being performed on inhabitants by the local government of Toyama prefecture and Ministry of the Environment Government of Japan.

Akita prefecture, located in the northern part of Japan, has many scattered cadmium-polluted areas because of the previous activities of many mines and smelters throughout the prefecture [4]. The whole width of cadmium-polluted areas, designated by the Anti-Farm Soil Pollution Law based on the production of rice with a concentration over the safety standard level, in Akita prefecture is similar to that in Toyama prefecture. Consequently, it has been estimated that farmers in these areas have been excessively exposed to cadmium and have developed cadmium nephropathy. Systematic health examinations were performed throughout Akita prefecture in the 1970s, resulting in the detection of 22 suspected patients with cadmium nephropathy out of approximately 3000 inhabitants in cadmium-polluted areas [8]. Of these, 13 suspected patients were from Kosaka town in the northern area, suggesting that it is the most polluted area in Akita prefecture. A large mine and smelter along with a few satellite mines were operated in this town. After systematic health examinations, two independent studies were performed in Kosaka town, which also identified patients with cadmium nephropathy [9, 10].

Between 2000 and 2004, decades after these studies were conducted, cross-sectional investigations on female farmers aged 40–79 years old were performed in two adjacent cadmium-polluted areas in Akita prefecture, one of which included Kosaka town and Kazuno city. Another area was Odate city, into which a river runs from Kosaka town, suggesting that its level of cadmium pollution was lower than that of Kosaka town [11]. Although farmers in both areas were excessively exposed to cadmium through the consumption of home-harvested rice, those in the area of Kosaka town were affected more, with women aged 70 years or older showing the significant deterioration of renal tubular function and one case of cadmium nephropathy being detected among them.

Only healthy people are investigated in health examinations, infirm individuals cannot participate, suggesting that patients with severe cadmium nephropathy or *itai*-*itai* disease may be overlooked. It is highly plausible that these patients are being treated at local medical institutions for renal dysfunctions of unknown causes based on elevated serum creatinine levels. Therefore, we postulate that cases of cadmium nephropathy may be detected among these patients in institutes by assessing renal tubular function in addition to cadmium exposure levels. In the present study, we examined patients who attended core medical institutes in Odate city and Kosaka town for the treatment of elevated serum creatinine levels by measuring urinary ß2MG as well as blood and urinary cadmium levels. This “hospital-based screening” is expected to effectively detect patients with cadmium nephropathy.

## Methods

### Selection of subjects

This hospital-based screening to detect cadmium nephropathy was performed in Akita Rosai Hospital in Odate city between 2010 and 2011 and Kosaka Clinic in Kosaka town in 2013, both of which are core institutes for regional medicine. In Odate, there were 1003 patients who attended the internal medicine of Akita Rosai Hospital in 2010. From them, we selected patients aged 60 years or older who lived in the cadmium-polluted area and showed elevated serum creatinine levels (0.8 mg/dL≤) and no definite renal diseases, resulting in 95 eligible ones. Then we asked them to participate in this study, obtaining agreement from 35 subjects. Following Odate, we asked outpatients in Kosaka who were aged 60 years or older with elevated serum creatinine levels (males 1.1 mg/dL≤, females 0.7 mg/dL≤) and no definite renal diseases sequentially, obtaining 22 subjects. After obtaining informed consent, heparinized peripheral whole blood and urine samples were collected. In addition, based on answers to a questionnaire, we confirmed information on, for example, their history of residence, rice consumption, and disease. We did not exclude smokers because the effects of smoking on blood or urinary cadmium levels are negligible in Japanese people with a highly accumulated level of cadmium [12].

### Handling and analyses of samples

Urinary samples were divided into three tubes: one drop of 0.1 mol nitric acid was added to the first tube to stabilize cadmium for the measurement of cadmium concentrations, one drop of 20% sodium carbonate was added to the second tube for the measurement of β2MG in order to prevent its destruction by low pH [2, 13], and the third tube had no additives for the measurement of creatinine and qualitative tests. After checking the qualitative tests, the second and third urinary samples were maintained at 4 °C and used in laboratory tests on the same day. Blood and urine samples for the measurement of cadmium concentrations were stored at − 20 °C or − 80 °C until analyzed. Urinary α1MG and β2MG, and urinary creatinine levels were assessed by the latex agglutination method [14] and an enzymatic method [15], respectively. Mitsubishi Kagaku Bio-Clinical Laboratories, Inc. (Tokyo, Japan) conducted all biochemical measurements. We adjusted urinary concentrations by creatinine.

### Measurement of cadmium concentrations in blood and urine

The blood and urinary cadmium concentrations were measured by using ICP-MS Agilent 8800 (Agilent Technologies, Tokyo, Japan) and AAS AAnalyst 800 (PerkinElmer, MA, USA), respectively. We measured urinary cadmium by AAS to avoid polyatomic interferences of molybdenum that is contained in urine in a large amount [16]. Before these analyses, samples were decomposed by a microwave with nitric acid and hydrogen peroxide and then dissolved in ultrapure water to be measured. For internal quality control of the blood cadmium determination by ICP-MS, we analyzed the standard reference material, BCR 634, BCR 635, and BCR 636 Human Blood provided by the Institute for Reference Materials and Measurements, European Union with certified cadmium values of 1.4 ± 0.4, 6.6 ± 0.6, and 11.6 ± 0.6 μg/L, respectively. We obtained BCR concentrations by the same measurement procedure as the blood samples taken from study subjects. The obtained cadmium concentrations for BCR 634, BCR 635, and BCR 636 were 1.60 ± 0.17, 7.61 ± 0.60, and 12.41 ± 0.39 μg/L, respectively, which were in agreement with the certified values. Likewise, urinary cadmium determination by AAS was assessed using SRM2670a High-Level provided by the National Institute of Standard & Technology, USA with certified cadmium value of 5.16 ± 0.11 μg/L. The obtained cadmium concentration was 5.23 ± 0.09 μg/L. The limit of quantification (LOQ) was the concentration equivalent to the signal of cadmium, which was equal to ten times the standard deviation of six repeated measurements of the blank signal. The values of blood and urine cadmium LOQ were 0.16 and 1.28 μg/L, respectively.

### Statistical analysis

Age and serum creatinine were presented as arithmetic means with ranges, while urinary α1MG and β2MG levels, and blood and urine cadmium levels were presented as medians with ranges, which did not follow a normal distribution. Values less than LOQ or the minimal measurable limit were replaced with half the value for the calculation.

## Results

We set criteria to detect cadmium nephropathy in reference to urinary β2MG levels for renal tubular dysfunction, and blood and urinary cadmium levels for the over-accumulation of cadmium. It is generally accepted that urinary β2MG levels of 1000–10,000 μg/g cr. and higher than 10,000 μg/g cr. indicate “irreversible tubular proteinuria” and “overt cadmium nephropathy,” respectively [17], and 10 μg/g cr. of urinary cadmium is the threshold to induce overt cadmium nephropathy [18, 19]. There is no established threshold of blood cadmium to induce cadmium nephropathy; therefore, we provisionally selected 6 μg/L in the present study based on the relationship between blood and urinary cadmium levels among 1464 female farmers in the same areas who were healthy examinees in a previous study [5], in which we selected 5.7 μg/L of blood cadmium (*y*) by substituting 10 μg/g cr. of urinary cadmium (*x*) into their regression equation: *y* = 0.396*x* + 1.692. Therefore, we diagnosed a subject with a urinary β2MG level higher than 10,000 μg/g cr. and blood cadmium level higher than 6 μg/L or urinary cadmium level higher than 10 μg/g cr. as having cadmium nephropathy.

Subject profiles in the two medical institutes are shown in Table 1. We enrolled 35 subjects (15 males, 20 females) from Odate and 22 (6 males, 16 females) from Kosaka, and their mean ages were approximately 80 years (range, 60–95 years). Urinary β2MG and blood and urinary cadmium levels in subjects showed very wide ranges and no significant relationships between them (data not shown), indicating the inclusion of subjects with renal tubular dysfunction and the over-accumulation of cadmium.

**Table 1:** Subject profiles in two medical institutes

	Akita Rosai Hospital in Odate city		Kosaka clinic in Kosaka town	
	Male	Female	Male	Female
Number	15	20	6	16
Age^a^	79.0 (61–88)	81.0 (60–95)	83.2 (73–92)	80.3 (71–89)
Serum creatinine (mg/dL)^a^	1.19 (0.84–2.82)	1.13 (0.80–2.05)	1.39 (1.24–1.67)	1.00 (0.71–1.90)
Urinary α1MG (mg/g cr.)^b^	6.8 (2.7–63)	17 (ND-94)	20 (7.9–37)	13 (4.9–62)
Urinary β2MG (μg/g cr.)^b^	249 (ND-14,700)	1570 (ND-34,000)	3160 (274–8290)	815 (394–30,400)
Blood cadmium (μg/L)^b^	3.2 (1.7–7.6)	4.7 (3.4–11)	4.3 (2.5–6.1)	4.6 (2.2–13)
Urinary cadmium (μg/g cr.)^b^	2.96 (1.24–9.54)	5.47 (1.59–11.2)	2.25 (ND-4.85)	5.08 (ND-16.2)

Data are presented as an arithmetic mean^a^ or median^b^ (minimum-maximum)

*α1MG* α _1_-microglobulin, *β2MG* ß_2_-microglobulin, *ND* not detected (β2MG, less than 70 μg/L as an original value; urinary cadmium, less than 1.28 ng/mL as an original value)

**Fig. 1:**
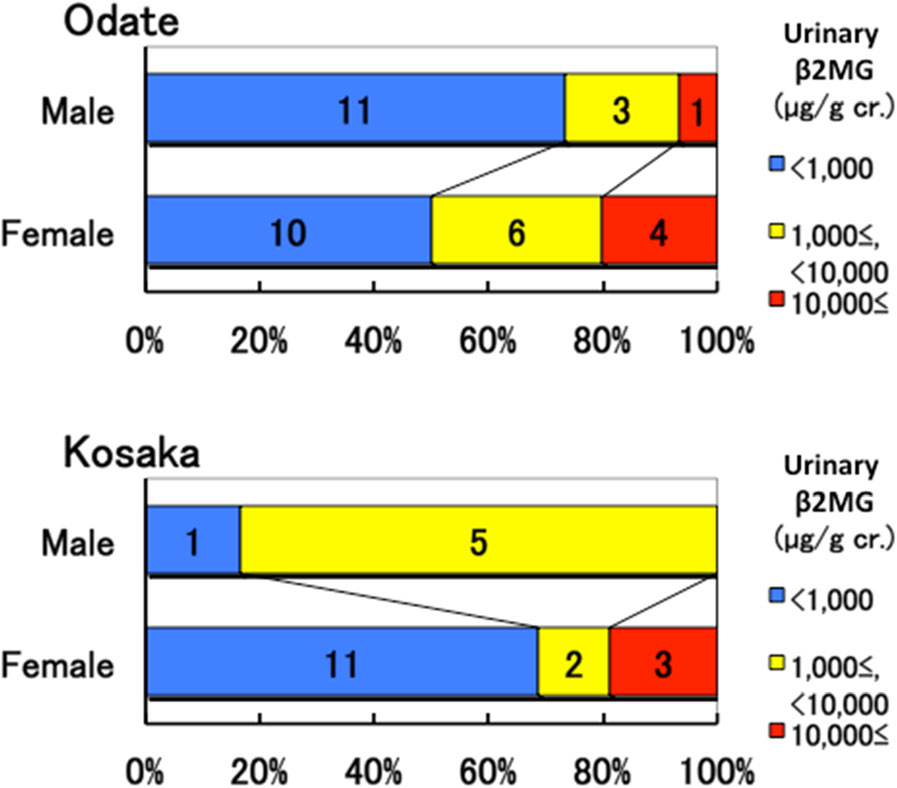
Distribution of urinary ß_2_-microglobulin (β2MG) levels among 35 and 22 subjects in Akita Rosai Hospital in Odate city (Odate) and Kosaka Clinic in Kosaka town (Kosaka), respectively. The actual numbers are presented in the bars

**Fig. 2:**
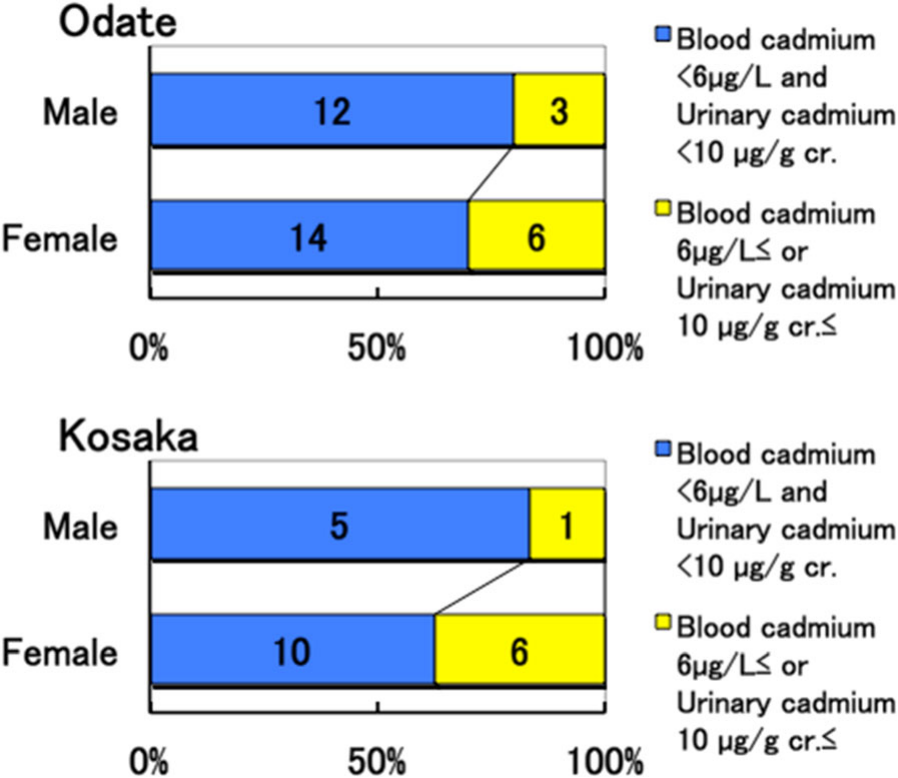
Distribution of blood and urinary cadmium levels among 35 and 22 subjects in Akita Rosai Hospital in Odate city (Odate) and Kosaka Clinic in Kosaka town (Kosaka), respectively. The actual numbers are presented in the bars

Therefore, we examined the distributions of urinary β2MG, blood cadmium, and urinary cadmium levels among subjects. Four out of 15 males (27%) and 10 out of 20 females (50%) in Odate, and 5 out of 6 males (83%) and 5 out of 16 females (31%) in Kosaka had urinary β2MG levels higher than 1000 μg/g cr. (Fig. 1). One out of 15 males (7%) and 4 out of 20 females (20%) in Odate and 3 out of 16 females (19%) in Kosaka had urinary β2MG levels higher than 10,000 μg/g cr. (Fig. 1). One male (5%) and seven females (19%) had urinary β2MG levels higher than 10,000 μg/g cr., and were suspected of having cadmium nephropathy. Three out of 15 males (20%) and 6 out of 20 females (30%) in Odate and 1 out of 6 males (17%) and 6 out of 16 females (38%) in Kosaka had blood cadmium levels higher than 6 μg/L or urinary cadmium levels higher than 10 μg/g cr. (Fig. 2). Four males (19%) and 12 females (33%) were at risk of cadmium nephropathy due to the over-accumulation of cadmium.

We then selected subjects with urinary β2MG levels higher than 1000 μg/g cr., blood cadmium levels higher than 6 μg/L, or urinary cadmium levels higher than 10 μg/g cr. as patients with the irreversible tubular proteinuria or the over-accumulation of cadmium (Tables 2 and 3). The values of urinary α1MG levels were presented along with them, which were in parallel with urinary β2MG levels. Sixteen and 12 subjects fulfilled each of the three conditions in Odate and Kosaka, respectively. Among them, one male and two females in Odate and three females in Kosaka were diagnosed with cadmium nephropathy, having urinary β2MG levels higher than 10,000 μg/g cr. and blood cadmium levels higher than 6 μg/L or urinary cadmium levels higher than 10 μg/g cr. All six patients had lived in Kosaka town, Kazuno city, or Odate city and consumed self-harvested or locally produced rice throughout their life. In addition, they had no history of occupational exposure to cadmium. Therefore, we detected 1 patient with cadmium nephropathy out of 21 male subjects, 5 out of 36 in female subjects, and 6 out of all 57 subjects, accounting for 4.8, 13.9, and 10.5%, respectively. There was one patient who was a participant in our previous health examinations in Odate, but none of the six patients with cadmium nephropathy participated in it.

**Table 2:** Subjects who fulfilled at least one of three conditions: a urinary β_2_-microglobulin (β2MG) level higher than 1000 μg/g cr., blood cadmium higher than 6 μg/L, or urinary cadmium level higher than 10 μg/g cr., out of 35 subjects in Akita Rosai Hospital in Odate city. The values of serum creatinine and urinary α_1_-microglobulin (α1MG) levels were also presented to support these data. They are listed in order of descending levels of urinary β2MG

No.	Sex	Age	Serum creatinine (mg/dL)	Urinary α1MG (mg/g cr.)	Urinary ß2MG (μg/g cr.)	Blood cadmium (μg/L)	Urinary cadmium (μg/g cr.)	Medical history of diagnosed patients
1	F	70	1.70	63.3	34,000	4.8	5.39	
2^a^	F	95	0.99	64.2	24,700	6.2	11.9	Hypertension, diabetes
3^a^	M	81	2.82	62.6	14,700	3.4	10.4	Diabetes
4	F	88	1.14	23.3	13,000	4.7	2.83	
5^a^	F	81	2.05	94.3	12,600	11	5.93	Unremarkable
6	F	84	1.22	40.1	7830	7.2	7.21	
7	F	73	1.10	29.3	7580	4.9	12.0	
8	F	74	1.24	35.7	6470	3.4	4.32	
9	F	60	1.02	47.3	5800	3.8	9.21	
10	F	86	0.88	21.0	3300	4.8	6.38	
11	M	79	1.07	11.9	3180	2.1	0.47	
12	F	81	0.80	15.9	2630	7.1	8.23	
13	M	87	1.60	11.9	2170	2.6	1.90	
14	M	80	0.86	17.1	1510	6.0	ND	
15	F	91	0.81	7.1	329	7.2	11.0	
16	M	78	1.01	8.1	249	7.6	8.46	

*F* female, *M* male, *ND* not detected (less than 1.28 ng/mL as an original cadmium level)

^a^Diagnosed with cadmium nephropathy

**Table 3:** Subjects who fulfilled at least one of three conditions: a urinary β_2_-microglobulin (β2MG) level higher than 1000 μg/g cr., blood cadmium level higher than 6 μg/L, or urinary cadmium level higher than 10 μg/g cr., out of 22 subjects in Kosaka clinic in Kosaka town. The values of serum creatinine and urinary α_1_-microglobulin (α1MG) levels were also presented to support these data. They are listed in order of descending levels of urinary β2MG

No.	Sex	Age	Serum creatinine (mg/dL)	Urinary α1MG (mg/g cr.)	Urinary ß2MG (μg/g cr.)	Blood cadmium (μg/L)	Urinary cadmium (μg/g cr.)	Medical history of diagnosed patients
1^a^	F	81	1.30	62.3	30,400	9.1	5.02	Hypertension, analgesic
2^a^	F	89	1.90	54.8	28,900	9.6	16.2	Hypertension
3^a^	F	75	0.98	43.3	14,800	13	5.23	Hypertension
4	M	84	1.29	34.9	8290	3.8	2.70	
5	F	81	0.89	29.4	5390	2.2	ND	
6	M	87	1.67	37.2	4960	6.1	ND	
7	M	92	1.24	7.9	3940	2.5	ND	
8	F	82	0.91	35.9	2770	6.9	8.01	
9	M	83	1.25	8.3	2380	4.8	ND	
10	M	80	1.46	26.5	1990	5.4	4.85	
11	F	77	1.44	19.8	963	7.6	10.9	
12	F	77	0.97	4.9	394	6.7	1.71	

*F* female, *M* male, *ND* not detected (less than 1.28 ng/mL as an original cadmium level)

^a^Diagnosed with cadmium nephropathy

## Discussion

Many cadmium-polluted areas exist not only in Japan but also worldwide, and health examinations for local inhabitants have been actively executed to investigate the adverse health effects of cadmium. However, to the best of our knowledge, hospital-based screening to identify patients with cadmium nephropathy in cadmium-polluted areas has not yet been conducted. We herein identified 6 out of 57 patients (10.5%) with cadmium nephropathy using this hospital-based approach. This detection rate is markedly higher than that of health examinations previously executed in the same areas, in which only 1 patient was identified among 1163 examinees [2]. These results indicate that this hospital-based screening is very effective for detecting patients with cadmium nephropathy in cadmium-polluted areas, and needs to be applied to other cadmium-polluted areas not only in Akita prefecture but also worldwide.

**Fig. 3:**
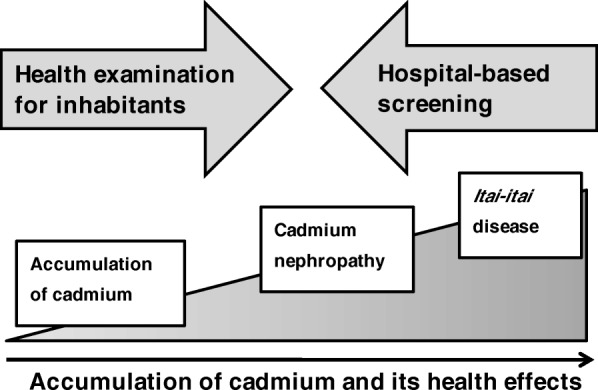
Schematic diagram of a health examination for inhabitants and hospital-based screening in cadmium-polluted areas to detect patients with cadmium nephropathy or *itai*-*itai* disease in relation to cadmium exposure levels and its health effects

Health examinations are useful for clarifying the overall status of cadmium exposure and its health effects among the local inhabitants of cadmium-polluted areas, and providing feedback to prevent further pollution or exposure to cadmium. Hospital-based screening functions in a complementary manner for local health examinations, detecting patients with advanced cadmium nephropathy or even *itai*-*itai* disease among those ineligible to participate in a health examination due to their poor physical condition. Finding these patients not only contributes to individual medical treatments for them but also further supports the result of health examinations to show the overall status of cadmium pollution in the areas. In other words, these two approaches with opposite directions may cover all phases in the natural history of cadmium toxicity, from low-level exposure to cadmium to *itai*-*itai* disease (Fig. 3). Therefore, local health examinations and simultaneous hospital-based screening in cadmium-polluted areas are strongly recommended.

In the present study, we measured urinary β2MG and blood and urinary cadmium levels in screening as indicators of renal tubular dysfunction and the over-accumulation of cadmium, respectively. However, these are the minimum requirements that were practically set in consideration of the operating conditions of these medical institutes. Although urinary β2MG is widely used as a good indicator of cadmium-induced renal tubular dysfunction because of its high sensitivity and wide range, it has the disadvantage of degrading under low pH [13]. Therefore, the measurement of other types of indicators of renal tubular dysfunction, such as α1MG and retinol binding protein, is additionally recommended. Actually, we measured urinary α1MG levels in this study, showing elevation in parallel with urinary β2MG levels to support the renal tubular dysfunction. Urinary cadmium is considered to reflect the level of cadmium that has accumulated in the kidneys; however, in an advanced phase of renal dysfunction, it decreases due to the destruction of renal tissue. In this case, blood cadmium is used as an indicator of the level of cadmium that has accumulated in the body [1]. Therefore, blood and urinary cadmium levels need to be simultaneously measured in populations with high-level exposure to cadmium.

We adopted the following criteria for cadmium nephropathy: a urinary β2MG level higher than 10,000 μg/g cr. and blood cadmium level higher than 6 μg/L or urinary cadmium level higher than 10 μg/g cr., which were relatively high because they were introduced from clinical observation on patients with overt cadmium nephropathy and *itai*-*itai* disease. Since these criteria were set to accurately diagnose cadmium nephropathy, they may be too strict for the purpose of screening. For example, in official health examinations for the inhabitants of the cadmium-polluted area of Toyama prefecture, the cut-off value for urinary β2MG was set to 5000 μg/g cr. Other than patients diagnosed with cadmium nephropathy, there were subjects with a urinary β2MG level sufficiently high to indicate irreversible tubular proteinuria or the accumulation of cadmium close to the threshold (Tables 2 and 3). It cannot be denied that these subjects may be developing cadmium nephropathy. Therefore, a pre-phase of cadmium nephropathy may need to be established in addition to the diagnosis of cadmium nephropathy and their health status may also need to be followed up, similar to diagnosed patients. Further investigations are warranted.

The results of this hospital-based screening indicate that appropriate medical care needs to be provided to patients diagnosed with cadmium nephropathy. For example, measurements of the tubular reabsorption of phosphorus (TRP) will further confirm the status of renal tubular dysfunction. Unfortunately, effective treatments for the renal damage of patients with cadmium nephropathy do not exist, but secondary osteomalacia and renal anemia can be prevented or treated. Since abnormal bone metabolism is expected, markers such as serum alkaline phosphatase also need to be assessed. X-ray photographs of bones may be important for detecting osteomalacia in patients suspected of having *itai*-*itai* disease. There are various kinds of medicine for abnormal bone metabolism available today. If patients present with anemia, the measurement of serum erythropoietin as well as markers of iron metabolism, such as serum iron and ferritin, is necessary for reaching a diagnosis and treating renal anemia using appropriate erythropoiesis stimulating agents [20].

There were several limitations to the present study. Its design was a case series study without controls. Since it currently remains unknown how many patients with elevated serum creatinine levels not related to cadmium exposure had ß_2_-microglobulinuria, difficulties are associated with accurately assessing whether the detection rate of ß_2_-microglobulinuria among patients with elevated serum creatinine levels in the institutes in cadmium-polluted areas was high. A hospital-based study generally has the disadvantage of the actual rate of disease not being known due to the lack of an exact number of the denominator. However, the primary purpose of the present study was to validate the efficacy of hospital-based screening to detect cadmium nephropathy, and not to clarify the detection rate. Further studies are needed to investigate the general rate of ß_2_-microglobulinuria among patients with elevated serum creatinine levels relative to those with cadmium nephropathy in medical institutes in cadmium-polluted areas. The ways of selecting subjects were different between Odate and Kosaka. Because we first began this study as a pilot study in Odate, the criteria of serum creatinine was set without gender difference for the convenience of selection. After getting hopeful results in Odate, we then set minute criteria of serum creatinine in Kosaka with gender difference. In the future, it would be better to use eGFR for the selection criteria. In addition, while we selected the subjects from all outpatients by narrowing-down in Odate, the subjects were selected in a random, sequential manner in Kosaka, where the number of all outpatients was much smaller. Although it may be ideal to set a strict procedure to select subjects, it is reasonable to adopt various selecting ways optimal to each medical institute. One patient with cadmium nephropathy had taken an analgesic for more than 11 years, which is known to have the side effect of inducing renal injury. Other than that, three patients had hypertension, one patient had diabetes, and one patient had both hypertension and diabetes, which might have affected the observed their renal function. Therefore, nephropathy in these patients may have been derived from the combined effects of cadmium exposure and these factors. Cadmium nephropathy is intrinsically proximal renal tubular dysfunction, but it gets accompanied by renal glomerular dysfunction at the end stage and shows an increase of serum creatinine. The hospital-based screening in this research is aimed at detecting such a patient with cadmium nephropathy at the end stage. However, many elder patients in a hospital often suffer from hypertension or diabetes or take medicines that might affect renal glomerular function. Therefore, although it might be difficult to differentiate the causes of renal glomerular dysfunction, it would be reasonable to judge as cadmium nephropathy based on the history of living in cadmium-polluted areas, extremely high levels of blood or urinary cadmium, and urinary ß2MG, which are too rare to be observed in a usual daily clinical practice. That is why the criteria of cadmium nephropathy were set at relatively high levels.

## Conclusions

Based on hospital-based screening in cadmium-polluted areas at which urinary β2MG and blood and urinary cadmium levels were measured, we effectively detected patients with cadmium nephropathy among patients with elevated serum creatinine levels without definite renal diseases. This hospital-based screening will play a complementary role in health examinations for local inhabitants in cadmium-polluted areas, and the simultaneous application of these approaches will lead to the detection of all phases of cadmium toxicity and the provision of appropriate medical care for these patients.

## Abbreviations

ß2MG: ß_2_-microglobulin
α1MG: α_1_-microglobulin
